# An innovation on clinical placement for occupational therapy mental health during the COVID-19: A mixed-methods feasibility study

**DOI:** 10.3389/fmed.2022.967511

**Published:** 2022-10-19

**Authors:** Farahiyah Wan Yunus, Muhammad Hibatullah Romli, Hanif Farhan Mohd Rasdi, Dzalani Harun, Masne Kadar

**Affiliations:** ^1^Center for Rehabilitation and Special Needs Studies, Occupational Therapy Programme, Faculty of Health Sciences, Universiti Kebangsaan Malaysia, Kuala Lumpur, Malaysia; ^2^Department of Rehabilitation Medicine & Medical Education Research and Innovation Unit (MERIU), Faculty of Medicine and Health Sciences, Universiti Putra Malaysia, Serdang, Malaysia

**Keywords:** psychiatry, education, fieldwork, simulation, clinical reasoning

## Abstract

The COVID-19 pandemic altered the health profession's education. Educational activities were shifted to online, and clinical placements were compromised in certain countries. A mixed-methods study included 17 undergraduates undergoing a mental health placement. The first 3 weeks of clinical placement applied online case-based learning in written and in video format. The last 2 weeks involved hybrid remote and physical onsite clinical placement. SPICES model utilizing various active learning activities, case studies and client attendance, facilitator engagement, discussion and feedback were implemented. A self-administered System Usability Scale (SUS), e-learning preference level, focus group discussion, and reflective writing was conducted at the end of each week and the students' final marks were compared with the past cohort who attended conventional physical clinical placement. Two-way mixed ANOVA indicates no significant interaction was found on the SUS (*p* = 0.062, $\eta_{\text{p}}^{2}$ = 0.062) and preference scores (*p* = 0.285, $\eta_{\text{p}}^{2}$ = 0.079) according to week and practical site. There was no significant difference in the final mark among the online and onsite placement of the current cohort (*p* = 0.350, d = 0.47). The current cohort reported better marks than the previous cohort who attended conventional placement (*p* = 0.006, d = 0.99). Qualitative findings show positive responses where online activities have minimal restriction on the learning process. This innovative approach is acceptable for substituting conventional clinical learning during this restricted situation.

## Introduction

The sudden interruption of the COVID-19 pandemic disrupted human daily life and activities. Measures to prevent the disease spread were taken by maintaining physical distance, face mask-wearing, and frequent maintenance of good hygiene practices, especially hand hygiene (1). Malaysia enforced movement restrictions to prevent and control the spread of the disease. Consequently, the Ministry of Higher Education had limited options and took action to halt on-campus learning and shift to fully online learning for the safety of the students (2). The alteration to online learning was supported internationally during this emergency period (3, 4). However, this disrupted the learning process and accelerated the transition from conventional to technology-based learning without ample time, resources, and space for preparation.

The shortage of healthcare professionals is alone a crisis, especially in developing countries (5). A delay in learning may defer in producing new healthcare practitioners. Therefore, the teaching and learning process needs to continue for the students to complete their studies. The initiative of strategic technology reliance such as e-learning during the pandemic was crucial. It has maintained the progress, minimized the impact, sustained the pace of learning activities, and supported the system survival (i.e., academic, employment) of a nation (6). Moreover, mental health practice has the challenge to recruit and retain enough practitioners; thus, ensuring students' interest in this practice is crucial (7). Nevertheless, this has brought a massive challenge to universities as educators needed to shift to full online teaching quickly.

Health professions' education heavily relies on hands-on training. Compared to other educational programs such as social sciences or other technological sciences, health professions education is majorly impaired by barriers and restrictions to fully online learning (8–10). The acceptance of technology to substitute many activities are also reported to be less desirable and creates conflict with many parties such as clients (i.e., patients), healthcare providers, educators, and even the public (10–12). Health sciences education, such as occupational therapy, relies on precision skill in many psychomotor aspects in managing patients to ensure best service and safety as the main priority while reducing any errors in performing health interventions (9, 12). This requires close observation and direct experience to be gained by the students. At the same time, immediate feedback from the educators, supervisors, or preceptors is warranted.

Online learning is consensually perceived as having limitations on skills development, primarily related to technical and psychomotor aspects (13, 14). However, several reviews indicated that learning *via* alternative methods such as video debriefing (15) and using clinical cases to aid teaching (a.k.a., case-based learning) (8) is beneficial to develop learners' clinical and soft skills. Using simulation as simple as voice simulation in mental health practice is valuable for skill learning (16, 17). Remote clinical learning is a valuable tool, especially during the COVID-19 period. However, several limitations still exist, such as technical issues, reduced engagement, and loss of assessments (18). There is also a concern among occupational therapy thinkers who opined online learning may not achieve the standard delivered by mainstream learning practice (12, 19, 20). The evidence on the effectiveness of alternative methods for clinical placement, such as case-based learning seems lacking in practice compared to physical setting clinical competency (21, 22) and is negligible in the occupational therapy arena. Hence, although there is a recommendation to substitute part of clinical placement with other alternative methods such as simulation and video learning, the effectiveness of the methods on clinical skills is relatively unknown. Therefore, an exploration of innovative clinical learning is required to ensure the quality of educational outcomes achieves the required standard.

## Methods

### Study design

This study adopted a quasi-experiment design, as participants were assigned in groups without randomization. A mixed-methods approach was used to evaluate the outcome of the intervention. The study utilized a longitudinal approach, collecting data on qualitative focus group discussion and quantitative survey at five-time points. The triangulation approach integrates the qualitative and quantitative findings (23).

### Participants

All undergraduate occupational therapy students in a single cohort who undergo clinical attachment for mental health placement were included in the study. The cohort was supposed to attend the clinical attachment at a physical setting–compulsory for the program requirement; however, due to the COVID-19 pandemic, the placement was put on hold. At the same time, access to the clinical setting was suspended. Nevertheless, the learning needs to continue to prevent any academic study postponements. Therefore, the institution's clinic was proposed as an alternative setting. However, the number of clients in the hospitals/clinics coming to seek treatment was also limited. Therefore, an innovation of flexible and dynamic learning activities was proposed, as permitted by the accreditation body for alternative methods application, as long as it fulfills the learning outcome purpose and can provide rich clinical learning experience to the students (4).

### Procedure

Although several model approaches (evaluative models, active models, active-evaluative models) are available in occupational therapy placement (24), this study selected an active-evaluative model to develop more comprehensive and holistic learning opportunities. Specifically, the SPICES model was implemented, emphasizing student-centric, problem-based, integrated teaching, community-based, electives, and systematic learning (25). Table 1 illustrates the learning strategies implemented with its reasoning addressing the SPICES requirement.

**Table 1:** The learning strategies implemented with its reasoning addressing the SPICES requirement.

**Spices**	**Learning strategy**	**Purpose**
Student-centric	•Team-based learning •Case-based learning •Problem-based learning	The use of strategies to foster active engagement and discussions among the learners.
Problem-solving	•Team-based learning •Case-based learning •Problem-based learning	The students initiative to creatively explore and search for resources from past learning material and internet for assessment and intervention plan.
Integrated knowledge	•Tutorial	The students approach the facilitator for advice and suggestions. The facilitator plays a role to ignite students to recall their memory on past learning.
Community-based	•Facilitator (lecturer and clinician) engagement	The students are encouraged to critically think about the clients/case intervention planning beyond that if conducted in a hospital setting and include the client's daily life activities.
Elective	•Discussion and feedback from peers and facilitator	To explore the advanced practice and creatively develop the alternative intervention.
Systematic	•Various case studies (written/video) •Real clients meeting (physical/virtual)	Provide a controlled exposure and rich learning experience to the students.

**Figure 1:**
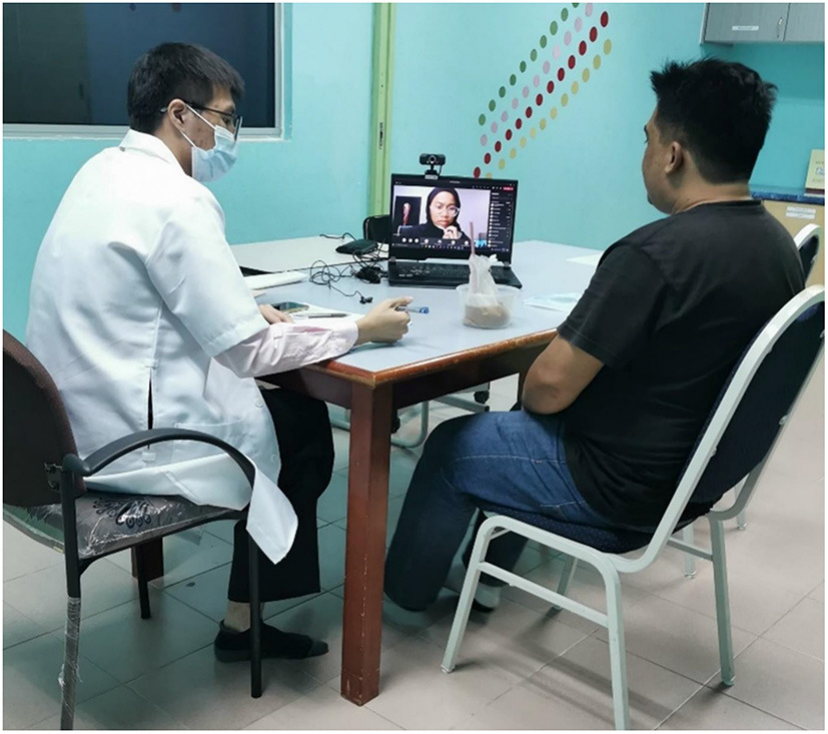
Example of clinical set-up onsite and online synchronous interview.

During the early briefing of this project, the lecturer explained the task the students were required to do, which is part of their learning process. The clinical practice is implemented for 5 weeks applying the concept of case-based and team-based learning. During the fourth week, half of the students were allowed to attend physical clinical placement with an actual client due to lockdown relief and permission given. The other half, viewed and participated in the clinical learning *via* virtual teleconference. There were 17 students in total and were divided into four groups. The virtual placement students participated synchronously with the physical placement student through the teleconference facility. The teleconference was performed using the Microsoft Teams application through a computer, laptop, or smartphone. The laptop/smartphone was placed at the corner of the table in the clinical setting to allow for an optimal view. The standard structure of the set-up is shown in Figure 1.

For this project, the clinical placement course coordinator–also a lecturer–acted as a facilitator for the activity. The clinician was also involved during the learning process. Standard one-week learning consists of 5 days. During the first 3 weeks, of the typical learning structure; students were provided with the initial information on several case studies, either in written or in video format. The written case studies were based on the real case previously completed. The initial information consists of the clients' sociodemographic information, diagnosis, medical, social, occupational history, etc. The initial information was uploaded in the clinical placement course's learning management system (LMS), which can be accessed by the students online. The LMS is standard for the institution's blended-learning concept, and the students are familiar with it. Next, the students independently discuss the case studies in their group, which consisted of history taking and possible assessments to be used, and potential intervention plans using any convenient platform. The facilitator was available during the session for the students to reach. The problem-based learning concept (26) was implemented at this stage.

Next, each group needs to present their discussion and demonstrate how to administer their choice of assessments. The facilitator gave feedback to each group presentation and then presented the outcomes and findings of the actual assessment from the case study. The students then discuss, compare their findings, and refine the intervention plan. Next, the students need to demonstrate how to perform the intervention based on their creativity. The presentation was presented using Microsoft PowerPoint. The students may use video, mock patient, self-demonstrate, or role-play among the group members using the teleconference system for the demonstration. Once again, the facilitator gave feedback for each group and then presented the actual intervention done for the cases. The students discuss and provide their feedback. A team-based learning technique (27) was implemented at this stage.

A similar approach was made for the video case study. Each group was given a different case-study video of an actual mental health scenario—the video depicting interview and assessments session among mental health clients and health practitioners. The video can be reached from the YouTube platform, and the video was for public purposes. The students were then required to discuss and present the strengths and limitations identified from the video. The students need to justify their argument, and any knowledge gap was then discussed later for learning issues activity. Next, the students were required to discuss potential assessments and interventions from the occupational therapy perspective for the video case study and do a presentation but without demonstrating. The facilitator provided feedback and suggestions for each group.

Each week, the students were allocated a day to complete reflective writing and a focus group discussion on their learning perception and experience for that week. The students were also required to answer the System Usability Scale questionnaire individually. The timetable for the 5 weeks of learning is illustrated in Table 2.

**Table 2:** Timetable for the clinical learning.

**Session day**	**Week 1**		**Week 2**		**Week 3**		**Week 4**		**Week 5**	
	**Morning**	**Afternoon**	**Morning**	**Afternoon**	**Morning**	**Afternoon**	**Morning**	**Afternoon**	**Morning**	**Afternoon**
Monday	Discussion on two written case studies (LMS and PBL)		Discussion on two written case studies (LMS and PBL)		Discussion on three written case studies (LMS and PBL)		Pair of remote (online) and physical clinic attendance with actual clients (Figure 1)	Small group discussion using horseshoe technique	Pair of remote (online) and physical clinic attendance with actual clients (Figure 1)	Small group discussion using horseshoe technique
Tuesday	Students' demonstration on decided assessment on the case studies and feedback from the instructor (TBL)	Students' demonstration on decided interventions on the case studies and feedback from the instructor (TBL)	Students' demonstration on decided assessment on the case studies and feedback from the instructor (TBL)	Students' demonstration on decided interventions on the case studies and feedback from the instructor (TBL)	Students' demonstration on decided assessment on the case studies and feedback from the instructor (TBL)	Students' demonstration on decided interventions on the case studies and feedback from the instructor (TBL)	Pair of remote (online) and physical clinic attendance with actual clients (Figure 1)	Small group discussion using horseshoe technique	Pair of remote (online) and physical clinic attendance with actual clients (Figure 1)	Small group discussion using Buzz group technique
Wednesday	Discussion on two written case studies (LMS and PBL)		Discussion on one video case study for each group (PBL)		Discussion on three written case studies (LMS and PBL)		Pair of remote (online) and physical clinic attendance with actual clients (Figure 1)	Small group discussion using Buzz group technique	Pair of remote (online) and physical clinic attendance with actual clients (Figure 1)	Small group discussion using horseshoe technique
Thursday	Students' demonstration on decided assessment on the case studies and feedback from the instructor (TBL)	Students' demonstration on decided interventions on the case studies and feedback from the instructor (TBL)	Discussion on two written case studies (LMS and PBL)	Students' demonstration on decided assessment and intervention on the case studies, and feedback from the instructor (TBL)	Students' demonstration on decided assessment on the case studies and feedback from the instructor (TBL)	Students' demonstration on decided interventions on the case studies and feedback from the instructor (TBL)	Pair of remote (online) and physical clinic attendance with actual clients (Figure 1)	Small group discussion using horseshoe technique	Pair of remote (online) and physical clinic attendance with actual clients (Figure 1)	Small group discussion using Buzz group technique
Friday	Reflection and portfolio writing; focus group discussion and survey		Reflection and portfolio writing; focus group discussion and survey		Reflection and portfolio writing; focus group discussion and survey		Reflection and portfolio writing; focus group discussion and survey		Clinical viva. Reflection and portfolio writing; focus group discussion and survey	

LMS, Learning Management System; PBL, Problem-Based Learning; TBL, Team-Based Learning.

For the last 2 weeks, nine students attended face-to-face clinical learning. The remaining eight students participated in the clinical learning *via* teleconference. As the allotment was promptly announced, the nine students attending clinic in person were divided into two groups. In comparison, the other eight students attending virtually were also divided into two groups, paired with their peers based onsite, and continued with online learning. The onsite students becomes representative for the online group *via* a buddy system, and the practice was conducted *via* teleconference. A clinician supervised the session. For the students who attended the physical clinic, they needed to adhere to the COVID-19 standard of practice; wearing complete personal protective equipment (i.e., mask, disposable gown, gloves), sanitizing hands before touching the client, and having close contact with clients only when necessary. As the number of clients was limited, rotation was made among students for each client to allow learning opportunities. At the same time, the other students observed the live session *via* Microsoft Teams. In the fourth week for the first 4 days, the morning session was reserved for clients attending, and the afternoon session was reserved for group discussion. On average, four clients attended the clinic every day.

The fourth week is allocated only for history taking, assessment, and intervention planning. The same clients came to the clinic in the fifth week, the intervention was implemented. The last day was reserved for clinical viva alongside reflective writing, focus group discussion, and the questionnaire.

### Setting

For actual clinical experience, the occupational therapy clinic at the institution was selected for the clinical placement. The clinic is a small-scale facility operated by four occupational therapists and one administrative staff. Clients were carefully selected and informed about the purpose of the clinical learning. The clients were mental health patients and consisted of psychosis and neurosis cases. The therapists contacted the clients *via* telephone and explained their expected involvement and role in the intervention and asked about their willingness to participate in the study. The participation of clients in the clinical session and this study was voluntary. The client was then scheduled for an appointment and the COVID-19 minimal standard operating procedure was explained. The client needed to wear a mask, sanitize their hands during the clinic session, declare that they are a low-risk for COVID-19 (e.g., absent of symptom, no contact with any COVID-19 cases, and not in COVID-19 contact-tracing list), have their temperature taken and record their attendance electronically. The client's consent was then taken manually, where the client needed to sign the consent form when they arrived at the clinic.

### Data collection

The data collection was naturally embedded in this course's learning activities and assessments. Therefore, the students were not significantly burdened by the research task as most activities formed part of the learning process and programme requirements. The assessment was formative and consisted of reflection (focus group discussion, which translated into reflective writing) and the mark for the course. While the students needed to complete the SUS questionnaire each week which was outside of their core learning activity, the questionnaire only took 10 min, and the burden was considered minimal.

#### Quantitative

Two outcomes were considered for the quantitative aspect to investigate the feasibility of the innovative clinical learning approaches. First, the System Usability Scale (SUS) questionnaire was administered every week. Specific terminology was adapted where needed to suit the study while maintaining the intended meaning of the question. The questionnaire needed to be completed online by the students individually. The SUS is a self-administered questionnaire containing ten items evaluating an individual's perception of the usability of the technology program. It is widely used internationally across the discipline, including health (28). Each item was scored using a five-point Likert scale, and the total score was calculated by adding up all item scorings. A higher score indicates a more positive perception of the technological approach. The SUS has extensive evidence supporting its positive validity and reliability (28). Second, a single-item question was developed to evaluate the students' preference on the online over traditional clinical placement by asking, “*I prefer this online clinical learning over traditional clinical placement*” and rated, with a 10-point Likert scale (1 = least prefer; 10 = most prefer). Using a single-item Likert gives less burden to the participant, is valuable, and still captures the meaningful outcome (29).

#### Qualitative–Group reflective writing

After the session, the students were required to write a group reflection and submit this into the LMS at the end of every week. The course coordinator was available during the allocated day so that students may ask for any clarification about the task. The students were provided with guiding questions but were not asked to follow these prescriptively. The research team reviewed the questions every week and updated them when necessary. The guided questions are listed in Table 3. In addition, the interpretation of the meanings was confirmed back with the students allowing credibility of the results. The credibility was conducted by online discussion at the beginning of the week after. The facilitator summarized each group's reflection and the students responded either the understanding is correct or requires further clarification. All students were informed that their participation is voluntary, majority of the students participated each week.

**Table 3:** Guided questions for the focus group discussion.

**No**.	**Questions**
1.	Why are you practicing clinical in an online mode?
2.	How do you feel about this new learning online clinical approach?
3.	How does this online clinical approach assist your learning and clinical skills?
4.	What are the advantages of practicing clinical skills online?
5.	What are the disadvantages of practicing clinical skills online?
6.	Are there any suggestions to improve the delivery of this online clinical?
7.	Do you prefer traditional clinical placement of going to the hospital or this online mode?
8.	How does online clinical learning differ from the usual traditional clinical placement/teaching?
9.	What are the difficulties you had during this new learning approach in this online clinical learning?
10.	What motivates you to use this online clinical approach in the future?
11.	Can you share with us the things that facilitate you in using this new learning approach?
12.	How equipped are you in participating in this online learning?

#### Retrospective comparison

The efficacy of the new approaches needs to be investigated to ensure if the approaches achieved the desired learning outcome. Therefore, the final mark for the clinical placement course was obtained from this current cohort and the previous year cohort who attended the conventional physical clinical placement to be compared. The comparison is possible as both cohorts implemented the same learning outcomes and assessments requirement as it is a standard according to the developed proforma and curriculum guideline. The learning outcomes are: (i) Identify basic management of Occupational Therapy through observation and practical experience, (ii) Conduct Occupational Therapy assessments and interventions for individuals with psychiatric disorders under supervision, (iii) Able to show a variety of communication skills appropriate to all level of society, client/colleagues in occupational therapy practice, and (iv) Demonstrate cooperative, professional, and ethical behaviors in occupational therapy practice. For both cohorts, students' marks came from various assessments such as clinical preceptor's report, short and long case study writing, case study presentation, portfolio (including reflection), logbook, and clinical viva. The students need to perform OSCE assessment with a real standardized patient. The student's final marks for this cohort were collected and compared with the previous cohort. The course coordinator was responsible for both the current and previous cohort and thus accessed the students' marks. A comparison of learning and assessment activities between the two cohorts is described in Table 4.

**Table 4:** Comparison of learning and assessment activities between cohorts.

	**Current cohort (*n* = 17)**	**Previous cohort (*n* = 19)**
**Learning activities**		
Learning nature	The majority on meaningful simulated learning	Experiential learning
Learning opportunity	The majority on controlled and virtual environment	The clinical environment on workplace-based learning
Structure	Online for 3 weeks utilizing team-based, case-based and problem-based learning and hybrid with real clients meeting for another 2 weeks.	Fully practice with real clients meeting.
Duration	200 h in 5 weeks span	200 h in 5 weeks span
Facilitator	Course coordinator/lecturer and institution's clinical instructors	External clinical preceptors at the setting.
**Assessment activities**		
Clinical preceptor's report	The institution clinical instructor	Completed students' performance reports. External clinical preceptors completed students' performance report
Short case	Rated by the course coordinator	Rated by the course coordinator
Long case	Rated by the course coordinator	Rated by the external clinical preceptor at the setting
Case study presentation	The presentation was marked by the coordinator/lecturer and external clinical preceptors.	The presentation was marked by the coordinator/lecturer and external clinical preceptors.
Portfolio	Self-writing and reflection by the students on their learning. Write summary/history of the settings. The marking was done by the coordinator/lecturer.	Self-writing and reflection by the students on their learning. Write summary/history of the settings. The marking was done by the coordinator/lecturer.
Logbook	Students need to obtain signatures from the institution's clinical instructors once they have completed each assignment (i.e., use of specific assessment and interventions)	Students need to obtain signatures from the external clinical preceptor at the setting once they have completed each assignment (i.e., use of specific assessment and interventions)
Clinical viva	A written case study was given, students' needs to demonstrate, role play and present the case, assessed by the coordinator/lecturers and the institution clinical preceptors.	Managing real clients and being assessed by coordinator/lecturer and external clinical preceptors.

### Data analysis

According to the practical site, two-way mixed ANOVA tests were conducted to determine the difference between SUS and preference across 5 weeks. The interaction effects (practical site^*^week) for both SUS and preference were presented to indicate whether SUS and preference scores had any significant differences (*p* < 0.05) according to practical site and week. The effect size for the analyses was also reported based on the partial eta squared values (small = 0.01, medium = 0.06, large = 0.14) (30). An independent *t*-test was also conducted to compare the significant difference in the final marks according to the practical sites. The effect size for the *t*-test was reported based on the Cohen's d (small = 0.20, moderate = 0.50, large = 0.80) (30). The qualitative data was analyzed narratively by summarizing students' reflective writing from week 1 to week 5. Students' direct quotes were coded into W1–5 for weeks 1 to 5 and G1–4 for groups 1 to 4. The qualitative data was analyzed chronologically to explore the progress on students' perception. The current situation is a new learning experience for the students and transition process is known to influence one's psychological condition which requires time to adjust (31). Both qualitative and quantitative findings are synthesized by complementing each other. Qualitative or quantitative findings may be used to confirm and explain each other, as both designs were conducted simultaneously (23).

## Results

All students (*n* = 17) consented to the data used in the study. There were more female (*n* = 13; 76.5%) than male (*n* = 4; 23.5%) in this study. In ethnic composition, majority were Malay (*n* = 8; 47.1%), and Chinese (*n* = 8; 47.1%) followed by Indian (*n* = 1; 5.9%). All students studied online for the first 3 weeks, while nine (52.9%) went for onsite placement during the fourth and fifth week, and eight (47.1%) remained online.

### Quantitative findings

Two-way mixed ANOVA tests were conducted to determine the difference between SUS and preference according to the practical site across 5 weeks. According to the analysis, there were no significant interaction effects of the week and practical site on the SUS and preference scores. There were no significant effects of the week and practical site on the SUS and preference scores for the main effects. The result are shown in Table 5. Therefore, it can be concluded that both SUS and preference scores had no significant differences according to practical site and week.

**Table 5:** Analysis of the SUS and preference level.

**Assessment**	**Practical site**	**Mean**±**SD**					**Interaction effect^a^ (p value)**	**Effect size^b^ ($\eta_{\text{p}}^{2}$ value)**
		**Week 1**	**Week 2**	**Week 3**	**Week 4**	**Week 5**		
System Usability Scale (SUS)	Onsite	34.78 ± 3.73	34.60 ± 4.36	34.94 ± 4.90	34.30 ± 2.93	33.82 ± 3.30	0.421	0.062
	Online	33.13 ± 2.64	33.78 ± 4.65	33.93 ± 3.84	30.93 ± 2.06	32.50 ± 4.31		
Preference	Onsite	5.33 ± 2.18	5.49 ± 2.47	3.84 ± 2.26	3.84 ± 2.64	3.84 ± 2.86	0.285	0.079
	Online	3.63 ± 1.85	3.84 ± 2.63	3.84 ± 2.50	3.84 ± 2.80	3.84 ± 3.27		

a Practical site^*^week; all main effects (i.e., practical site and week) for both SUS and preference scores are non-significant.

b Partial eta squared (small = 0.01, medium = 0.06, large = 0.14) (30).

Independent *t*-test showed no significant difference in the final mark between the two groups: full online (mean = 84.50, SD = 3.66) and online with physical onsite (mean = 82.89, SD = 3.22) in this current cohort (*p* = 0.350, d = 0.47). This indicates that the students may achieve similar performance to physical clinical attachment even though learned remotely.

However, a difference was found when the final mark, where the current cohort who learned online (*n* = 17; mean = 83.65, SD = 3.43) was better than the previous cohort who did a full onsite placement (*n* = 19; mean = 80.00, SD = 3.90), and the difference is significant (*p* = 0.006, d = 0.99). This indicates that structured and controlled clinical learning is not inferior and can nurture more active and meaningful learning than traditional clinical placement.

### Qualitative findings

Qualitative findings were reported chronologically to observe the changes in students' perception over time. The transformation of students' perception trend from negative to positive were identified.

### Theme 1: Skepticism of online clinical learning

Since this was the first time online clinical learning was implemented, students were pessimistic and confused about the implemented method.

“*We feel a bit confused as this is the first time, we try to make this online clinical approach and actually, do not have an idea on how the clinical placement can be done via online,”* W1G1.

Other students also felt awkward initially and were not looking forward to the newly introduced approach, especially during the first week of online clinical placement.

“*The new online clinical approach was awkward on the first day as some of us were confused on what to do,”* W1G2.

Even though the students found it difficult to follow during the first week of clinical placement, they still showed their enthusiasm to take part and were committed to learning.

“*We learnt a lot from every case study given. We imagined ourselves to be in real situations and helped each other to learn conducting the sessions. It was a good exposure for us since we were not able to have physical clinical placement. It was something new for us but we were enjoying it a lot,”* W1G3.

### Theme 2: Transition to the new method

Students' perception of online clinical learning began to change in week two after experiencing week one. The students began to adapt themselves to the new method introduced.

“*We are getting used to it [online clinical] and are willing to put more effort for every work that has been assigned,”* W2G2.

Students became more creative and enjoyed the assigned activity after experiencing the first week of online clinical.

“*We also enjoyed planning all of the flows and sequences of daily presentation to make it fun*,” W2G3.

Students also described the multiple learning experiences and the variety of exposure learned during the second week.

“*We learned quite a lot of interventions and some important professional ethics that we need to take note of during the interview session,”* W2G4.

In the second week, other videos on psychiatric cases were also discussed, and feedback from students was positive.

“*In this new learning online clinical approach, we found the videos that were assigned to us this week to be very informative and insightful. It gave us a clearer representation of the psychiatric clients, and the video also taught us more on the do's and don'ts while interviewing a real psychiatric patient,”* W2G2.

### Theme 3: Achieving the desired competency

Students also commented that they were ready and equipped with clinical skills in week three of online clinical learning. Feedback and comments immediately after each presentation helped the students understand the cases better and better understand the suitable interventions for each unique case.

“*We think that we are much more equipped in this online clinical placement for week 3. This is because studying and discussing long case studies gives us more opportunities to study the real case holistically. Comments and feedback from lecturers and clinicians also help us understand what interventions are appropriate to clients,”* W3G1.

One of the advantages students highlighted is that they could adapt and learn better with the help of peers and clinicians.

“*We could have more experiences and slowly adapt in presenting long case studies, frame of references, expose to more “extraordinary” cases as well as learn how to help them from our friends and clinicians,”* W3G4.

### Theme 4: Challenges arises in the transition

Students began to experience real clients for placement in the fourth and fifth weeks. Minor conflicts were aroused when adjusting to the new scenario. Comments of some of the challenges faced from the fully online students were:

“*Miscommunication or misunderstanding might happen as we could not hear the patient perhaps because of poor sound quality and a poor internet connection,”* W4G3.

They commented this was a bit of a burden for onsite students to set up the online equipment before the clinical session.

“*We need to set up all the online equipment and contribute much work to the whole clinical session with other friends,”* W4G2.

Even though there were challenges faced during the fourth week, the onsite students did try their best to assist their online peers. This is to ensure that their peers can gain the same experience as those onsite.

### Theme 5: Acceptance and compromising with peers

In the fifth week, the students were used to setting up and giving them the privilege of assisting their online friends.

“*It is because they are always [classmates] very cooperative and give support during the sessions. We all managed to provide the best to our online friends to get the same skills and knowledge just like us. Even though there are some flaws, our classmates adapt to the online learning method,”* W5G1.

The students were able to accept the online clinical learning well. In addition, the students also mentioned that the method was a great approach that can be implemented in the future.

“*The knowledge and skills learnt throughout this online placement had been our greatest motivation to apply this approach in the future. All of us agreed that this online clinical approach would be the best option for conducting sessions in the future. Our friends, clinician and Dr [the lecturer] had helped us a lot in using this new learning approach. Coming to the final week, all of us were more equipped for the placement and we felt more confident to plan and conduct the interventions with patients by applying the knowledge and skills learnt previously,”* W5G3.

Overall, students still preferred traditional physical placement in the hospital. They believed they will have better exposure and more learning experience in a natural setting with various cases. The students felt they did not have sufficient experience conducting assessment and intervention within the limited time [2 weeks only with real clients]. Students did comment that one of the benefits of online clinical is that they can record the session and monitor again if there is any unclear information. In addition, using Microsoft Teams, other students who do not have any client's appointment during that particular time can observe their friends conducting the assessment/intervention online without disrupting the session. On the positive side, the first three weeks of case study/video/ discussion activities did prepare them better to meet clients in the fourth and fifth weeks.

## Discussion

The current study reported that online learning for clinical learning and placement could achieve the targeted educational outcome. The learners were receptive and adapted to such contemporary learning. Although there were differences in the outcomes among the onsite and in-person groups on the learning system at week four, the difference was quickly diminished by the following week. A similar trend was noted on the preference level. Perhaps, this is because the learners, especially the remote ones, have a conflicting feeling when they first see their peers attend physical onsite clinical learning.

However, the satisfaction was relatively low. This is similar to the previous studies where the learners still prefer conventional learning (32, 33). The students in this study appreciated the effort given by the institution to continue the learning and considered the alternative clinical learning experiences as valuable despite the COVID-19 pandemic. However, students felt that online clinical learning did not provide a true clinical experience within an actual hospital environment and access to the full range of clients' diagnoses and behaviors. These findings reflect those reported elsewhere, particularly regarding the richness and realism of the learning experience (14, 32, 34). Undergoing full online learning is a new experience which contributes toward uncertainty and adjustment stress (14, 35). The challenges found in this study also reflected those in previous studies such as lack of facilities (e.g., poor internet connection), lack of teaching and learning preparation (inadequate learning resources), inconducive learning environment, and new task and burden (e.g., equipment set up, assignments) can also contribute toward low satisfaction (35). However, studying physically, such as on-campus and clinical attachment, is no better during this pandemic. Anxiety and worry about the COVID-19 significantly impact the learning process (36). Hence, similar to the previous study (37), the current study shows that learners are adapted and receptive to the new practice.

Although the learners perceived physical onsite learning as superior for their development, the findings indicated the plausibility of imitation and online learning to have a similar or even better outcome than physical clinical attachment. Previous literature supported the notion that alternative, online, and remote clinical learning can successfully achieve the learning objectives and may be greater than traditional clinical learning (15, 18, 21, 38, 39). An explanation for this is that the students have more opportunities to think, reflect, and receive feedback on their clinical practice critically. The ample time available for the students to discuss and come out with more precise decisions on assessments selection and creative intervention ideas, while the availability of the educator to provide immediate feedback for validation on the decision made and rectification on any misconception is helpful for learning. This quality of having the opportunity for reflection and reasoning is critical for success in clinical practice (8, 40). A supportive environment with positive educator quality catalyzed better clinical learning for the students (40). Proper teaching plan based on well-thought instructional design, utilizing learning theories, effective learning strategies, balancing facilitators involvement and student-centered learning is found to be beneficial (24). Clinical preceptors and instructors were found to have limited skills in clinical teaching, which makes the learning in traditional clinical settings less efficient (41, 42). In addition, learners perceived that they received little guidance in a clinical setting, which is less helpful for their learning (43). Thus, educators must be adequately equipped with education knowledge to scaffold the benefits of learning opportunities created.

Moreover, controlled learning, such as the online and use of case simulation, provides a safe and non-threatening environment for the students to explore and make an error for learning purposes. Such an environment was claimed to be helpful to facilitate the learners to be more active and engaged in learning (15, 18, 21, 38, 44). Using simulation, even the low fidelity (i.e., paper-based), is beneficial to developing the clinical skills (8). Furthermore, the traditional clinical setting is stressful due to high workload, lack of confidence in dealing with real patients, susceptible to errors and mistakes, intimidating environment, and risk on medico-legal issues, creating a less conducive environment and anxiety to the learners (44). Hence, applying evidence-based learning approaches such as case-based, problem-based, and team-based learning is argued to provide more meaningful learning and better outcomes (8, 45–47). This current study has presented as such. Thus, this study may open to a new approach for clinical learning to happen. During the pre-training, the purpose of clinical learning is not to make the learners highly competent in the clinical skills but to expose and equip the learners with the necessary clinical skills and reasoning. Hence, the skills can be nurtured after they graduate.

## Limitation

This current study was only limited to mental health clinical practice. Practice in mental health is more flexible than physical disciplines, which may need to follow a more rigid treatment procedures, require hands-on or physical contact, and require specialized skills development and competency (16, 17). Therefore, the transferability of the approach into other clinical settings is unknown. In addition, limited sample sizes may affect the study's findings, and larger sample sizes may contribute to different findings.

Although the assessment has been conducted by practicing a high level of integrity among the assessors and having standardized marking rubrics, assessments were not blinded thus may be susceptible to particular bias. Hence, the high involvement of the researcher in this study may unintentionally contribute toward observer bias on the favorable outcome or known as positive bias; Bygren (48, 49) better marks in the current cohort may be influenced by this. However, awareness of risk of being bias may also have made the assessors stricter, known as negative bias (48, 49). Hence, the findings from this study are valuable but need to be cautiously accepted.

## Future recommendation

A more rigorous methodology should be implemented. A true experimental design such as a randomized controlled trial (50) with larger sample size is required to investigate the effectiveness of this new clinical learning approach. Blinding of the assessors is desirable (48). Future studies may apply this new approach on different areas of clinical practice such as in pediatric, neuro rehabilitation and physical placement.

Implementing an actual practical placement *via* online application, with occupational therapists at the hospitals as facilitator can be explored. Therefore, students can gain better learning experience in a real live setting by interacting, evaluating and conducting interventions with the real client with guidance and assistance from the therapists. Transferring real clinical placement into technology application such as telehealth method has gained interest and attention in health profession education in recent years (51–53). This method may provide richer learning experiences.

## Conclusion

The COVID-19 situation has altered educational practice, including occupational therapy clinical placement. However, education needs to continue amidst the pandemic but requires to achieve high-quality standards. The relief on the clinical placement requirement with alternative substitution has created an innovative structure using the case-based, problem-based, and team-based learning approach through online learning as well as hybrid remote-physical onsite clinical learning with real clients. Such innovation was found to be effective in developing clinical reasoning and skills among the students comparable to conventional physical clinical learning.

## Data availability statement

The raw data supporting the conclusions of this article will be made available by the authors, without undue reservation.

## Ethics statement

The studies involving human participants were reviewed and approved by Research Ethics Committee of Universiti Kebangsaan Malaysia (Ref. No. JEP-2021-908). The patients/participants provided their written informed consent to participate in this study. Written informed consent was obtained from the individual(s) for the publication of any potentially identifiable images or data included in this article.

## Author contributions

FWY developed the study concept, conducted the data collection, and drafted the manuscript. MHR drafted the manuscript and performed the data analysis. All authors took part in result interpretation, reviewed and edited several versions of the manuscript, provided critical revisions, and approved the final version of the manuscript for submission.

## Conflict of interest

The authors declare that the research was conducted in the absence of any commercial or financial relationships that could be construed as a potential conflict of interest.

## Publisher's note

All claims expressed in this article are solely those of the authors and do not necessarily represent those of their affiliated organizations, or those of the publisher, the editors and the reviewers. Any product that may be evaluated in this article, or claim that may be made by its manufacturer, is not guaranteed or endorsed by the publisher.
